# Supplementary material for: Quantifying the Differences between 3D Virtual Planning and Attained Postoperative Reduction on CT for Patients with Tibial Plateau Fractures; a Clinical Feasibility Study
Source: J Pers Med. 2023 May 1;13(5):788. doi: 10.3390/jpm13050788 (PMC10222959; doi:10.3390/jpm13050788)
Supplement: Supplementary file 1 [file jpm-13-00788-s001.zip › jpm-2301825-Supplementary.pdf]

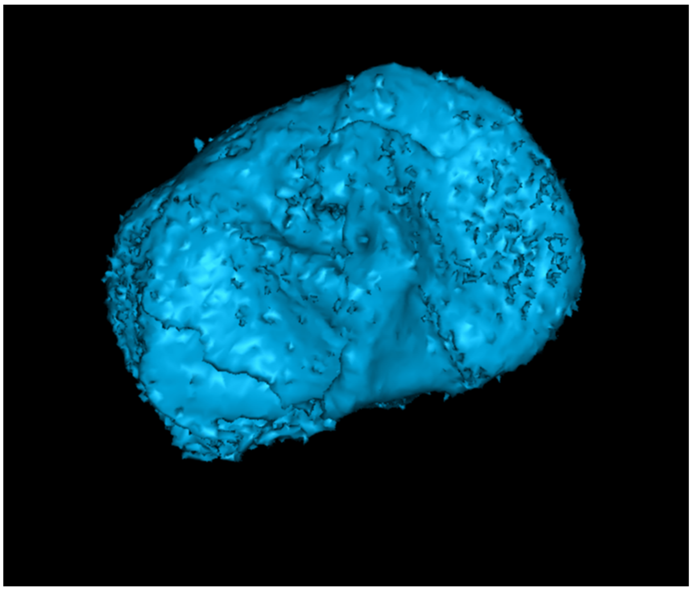

Video S1. Overview of one patients' differences between postoperative CT and 3DVP—plateau view**.**

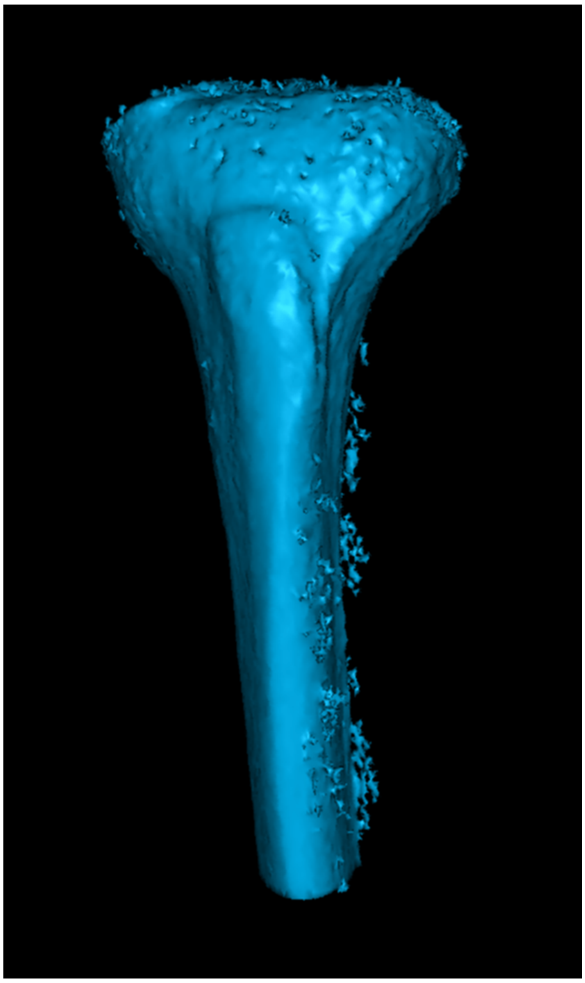

Video S2. Overview of one patients' differences between postoperative CT and 3DVP—AP view.
